# A non-linear beta-binomial regression model for mapping qlqc-30 to the eq-5d in lung cancer patients: a comparison with existing approaches

**DOI:** 10.1186/1745-6215-14-S1-O23

**Published:** 2013-11-29

**Authors:** Iftekhar Khan, Shah-Jalal Sarker, Steven Morris

**Affiliations:** 1 University College London, London, UK; 2 University of London - Queen Mary, London, UK

## Background

The performance of the Beta Binomial (BB) model is compared with several existing models for mapping the QLQC-30 on to the EQ-5D using data from lung cancer trials.

## Methods

Data from 2 separate non-small cell lung cancer clinical trials (TOPICAL and SOCCAR) are used to develop and validate the BB model. Comparisons with Linear, TOBIT, Quantile, Quadratic and CLAD models were carried out. The mean prediction error, R^2^, proportion predicted outside the valid range, clinical interpretation of coefficients, model fit and estimation of Quality Adjusted Life Years (QALY) are reported and compared. Monte-Carlo simulation from mixture distributions was performed to assess the performance of the models.

## Results

The Beta-Binomial regression model performed ‘best' among all models. Estimates from the BB were more accurate for predicting EQ-5D and QALYs compared to other modelling approaches. Mean difference in QALYs (predicted vs. observed) were 0.053 vs. 0.051 for TOPICAL and 0.162 vs. 0.164 for SOCCAR. Simulated 95% confidence intervals showed that the BB model contained the observed mean more often compared to the other models. All algorithms over-predict at poorer health states but the BB model was relatively better, particularly for the SOCCAR data.

## Conclusion

The Beta Binomial regression may offer superior predictive properties compared to existing algorithms and could be a more appropriate algorithm to map the relationship between the EQ-5D and QLQC-30. Future models could include toxicity data jointly with EQ-5D to improve prediction at poorer health states. The generalized lambda distribution may offer a way to simulate from a mixture distribution.

